# Effects of forest environment (Shinrin-yoku/Forest bathing) on health promotion and disease prevention —the Establishment of “Forest Medicine”—

**DOI:** 10.1265/ehpm.22-00160

**Published:** 2022-11-01

**Authors:** Qing Li

**Affiliations:** Department of Rehabilitation Medicine, Graduate School of Medicine, Nippon Medical School, Tokyo, Japan

**Keywords:** Blood pressure, Forest bathing, Forest Medicine, Immune function, NK, Nervous system, Phytoncide, POMS, Shinrin-yoku, Stress hormone

## Abstract

Humans have enjoyed forest environments for ages because of the quiet atmosphere, beautiful scenery, mild climate, pleasant aromas, and fresh, clean air. In Japan, since 2004, serial studies have been conducted to investigate the effects of forest environments (Forest bathing/Shinrin-yoku) on human health. My research team has established a new medical science called Forest Medicine. The Forest Medicine is a new interdisciplinary science, belonging to the categories of alternative medicine, environmental medicine and preventive medicine, which studies the effects of forest environments (Forest bathing/Shinrin-yoku) on human health. It has been reported that Forest bathing/Shinrin-yoku has the following beneficial effects on human health:

1 Shinrin-yoku increases human natural killer (NK) activity, the number of NK cells, and the intracellular levels of anti-cancer proteins, suggesting a preventive effect on cancers.
2 Shinrin-yoku reduces blood pressure and heart rate showing preventive effect on hypertension and heart diseases.
3 Shinrin-yoku reduces stress hormones, such as urinary adrenaline and noradrenaline and salivary/serum cortisol contributing to stress management.
4 Shinrin-yoku increases the activity of parasympathetic nerves and reduces the activity of sympathetic nerves to stabilize the balance of autonomic nervous system.
5 Shinrin-yoku improve sleep.
6 Shinrin-yoku increases the levels of serum adiponectin and dehydroepiandrosterone sulfate.
7 In the Profile of Mood States (POMS) test, Shinrin-yoku reduces the scores for anxiety, depression, anger, fatigue, and confusion, and increases the score for vigor, showing preventive effects on depression.
8 Shinrin-yoku may apply to rehabilitation medicine
9 Shinrin-yoku in city parks also has benefits on human health.
10 Shinrin-yoku may have preventive effect on COVID-19 by boosting immune function and by reducing mental stress.

Taken together, these findings suggest that Shinrin-yoku may have potential preventive effects on non-communicable diseases.

## What is Shinrin-yoku/Forest bathing?

Humans have enjoyed forest environments for ages because of the quiet atmosphere, beautiful scenery, mild climate, pleasant aromas, and fresh, clean air. Researchers in Japan have tried to find preventive effects against non-communicable diseases from forests and have proposed a new concept called “Shinrin-yoku/Forest bathing” [1–3].

Shinrin-yoku is translated into Forest bathing in English. Shinrin in Japanese means ‘forest’, and yoku means ‘bath’. Therefore, Shinrin-yoku means bathing in the forest atmosphere, or taking in the forest through our senses. This is not exercise, or hiking, or jogging. It is simply being in nature, connecting with it through our sense of sight, hearing, taste, smell and touch. Shinrin-yoku is like a bridge. By opening our senses, it bridges the gap between us and the natural world [1, 2].

People can enjoy the Shinrin-yoku through all five senses [3].

1. Sense of sight: green color, yellow color and red color, forest landscape, etc.
2. Sense of smell: special good smell, fragrance from trees and flowers, phytoncides.
3. Sense of hearing: forest sounds, listen to the birds singing and the breeze rustling in the leaves of the trees.
4. Sense of touch: touching trees, put your whole body in the forest atmosphere.
5. Sense of taste: eating foods and fruits from forests, taste the fresh air in forests.

## Why forest bathing/shinrin-yoku is necessary?

Stress is a keyword to understand why Shinrin-yoku is necessary in Japan. In 1984, the word ‘technostress’ was coined to describe unhealthy behaviour around new technology. Technostress can arise from all manner of everyday usage, like checking your phone constantly, compulsively sharing updates and feeling that you need to be continually connected. Symptoms run from anxiety, headaches, depression, mental fatigue, eye and neck strain to insomnia, frustration, irritability and loss of temper [4]. Since the year 2000, we have officially become an urban species. The urban population worldwide grew from just 746 million in 1950 to 3.9 billion in 2014, according to the United Nations Population Division. By 2050, 75% of the world’s projected 9 billion population will live in cities [2]. In Japan, prevalence of cancers and non-communicable diseases such as heart disease, diabetes, cerebrovascular disease and hypertension are increasing [5]) and more than half of deaths are attributed to non-communicable diseases [6]. According to the Ministry of Health, Labour and Welfare of Japan, the percentage of workers with anxiety and stress was more than 50% in 1982, 62.8% in 1997, 58% in 2007, and 60.9 in 2012 [3], suggesting a major mental health problem. Stress can induce almost all non-communicable diseases, such as cancers, hypertension, depression, cardiovascular diseases, stroke, gastric ulcer, obesity, alcoholism, panic disorder, eating disorder [7]. Therefore, the health management of workers, especially in relation to stress-related diseases, has become a major social issue and an effective new method for prevention of diseases is needed. There is also the phenomenon known as *karoshi*, or death from overwork in Japan. In 2016, the Ministry of Health, Labour and Welfare of Japan released a Cabinet-endorsed white paper on the extent of working overtime in Japan. Almost 23 per cent of companies said their employees worked more than eighty hours of overtime a month. Of those companies, 11.9% said some employees worked more than a hundred hours of extra time a month [2]. It is urgent to establish preventive measures against stress and non-communicable diseases; however, effective prevention methods have not been established. The forest environment has long been enjoyed for its quiet atmosphere, beautiful scenery, calm climate, clean fresh air and special good smell. Empirically, forest environments may reduce stress and have a relaxing effect; therefore, walking in forest parks may have beneficial effects on human health. Based on the above background, in Japan, a national health programme for Forest-bathing or Shinrin-yoku began to be introduced in 1982 by the Forest Agency of Japan for the stress management of workers in Japan. In 2005, my research team conducted the first Shinrin-yoku study in Iiyama, Nagano prefecture in Japan and the terms of Shinrin-yoku and Forest Bathing in English were first named and defined by author in this study [1]. Shinrin-yoku is also a short leisurely visit to a forest field, which is similar in effect to natural aromatherapy, for the purpose of relaxation and the breathing in of volatile substances called phytoncides (wood essential oils) derived from plants (trees), such as alpha-pinene and limonene [1, 8–10]. Because forests occupy 67% of the land in Japan, Shinrin-yoku is easily accessible [11]. It has become a recognized relaxation and/or stress management activity in Japan. Shinrin-yoku as a method of preventing diseases and promoting health is becoming a focus of public attention. According to a public opinion poll conducted in Japan in 2003, 25.6% of respondents had participated in a Shinrin-yoku trip, indicating its popularity in Japan [3]. Currently, the terms of “Shinrin-yoku” and “Forest bathing” are internationally accepted because both “Shinrin-yoku” and “Forest bathing” are the titles of English books [2, 12] and books in other languages [13, 14].

## What is Forest Medicine?

Imagine a new medical science that could let you know how to be more active, more relaxed, healthier and happier with reduced stress and reduced risk of non-communicable diseases. and cancers by visiting forests. This new medical science is Forest Medicine. Forest Medicine studies the effects of forest environments on human health and is a new interdisciplinary science, belonging to the categories of alternative medicine, environmental medicine and preventive medicine [3]. Forest Medicine is developed from Forest bathing or Shinrin-yoku and forest therapy and is an evidence-based preventive medicine. Forest Therapy is also developed from forest bathing (shinrin-yoku), which is a research-based healing practice through immersion in forest environments with the aim of promoting mental and physical health and improving disease prevention while at the same time being able to enjoy and appreciate the forest. Forest therapy is defined as a proven Shinrin-yoku effect (https://www.fo-society.jp/).

## Why forest medicine is necessary?

In Japan, a national health programme for Shinrin-yoku was proposed by the Forest Agency of Japan in 1982 for reducing stress in workers and the promotion of human health. However, when people started to practise Shinrin-yoku, in the early 1980s, it was based only on common sense and the intuitive idea that being in the beautiful green forests of Japan would be good for us. There has not been sufficient medical evidence supporting the beneficial effects of Shinrin-yoku due to technical limitations regarding measurements, and evidence-based evaluations as well as a therapeutic menu of Shinrin-yoku have been requested. Against this background, the Japanese Society of Forest Therapy was established in 2004 (https://www.fo-society.jp/) for conducting the evidence-based research on the effects of forest environments on human health. The Ministry of Agriculture, Forestry and Fisheries of Japan initiated a research project between 2004 and 2006 and organized a project team to investigate the therapeutic effects of forests on human health from a scientific perspective. In addition, recent technological developments have enabled us to determine the effects of forest environments on human health. Some people study forest, some people study medicine, I study forest medicine to find the beneficial effects of Shinrin-yoku on human health.

## Evidence-based Forest Medicine

In Japan, since 2004, serial studies have been conducted to investigate the effects of forest bathing/shinrin-yoku (forest environments) on human health by the project team. My research team has obtained a vast amount of data, proving that forest bathing promotes both physical and mental health by reducing stress [3].

### 1. Effects of Shinrin-yoku on immune system

It is well known that immune system including natural killer (NK) cells plays an important role in defense against bacteria, viruses and tumors. It is also well known that stress inhibits immune function. Forest environment (Shinrin-yoku/forest bathing) may reduce stress. Therefore, the author speculated that forest environment may have beneficial effect on immune function by reducing stress [3]. Thus, my research team conducted several experiments to investigated the effects of Shinrin-yoku on human immune function.

#### 1-1 Effect of Shinrin-yoku on human NK activity in male subjects

In the first Shinrin-yoku study [1], 12 healthy male subjects, aged 37–55 years, were selected from three large companies in Tokyo, Japan. The information of the subjects gathered from a self-administered questionnaire, including age and lifestyle habits. None of the subjects had any signs or symptoms of infectious diseases, used drugs that might affect immunological analysis, or were taking any medication at the time of the study. The subjects participated in a three-day/two-night trip to forest areas at Iiyama in Nagano prefecture located in the Chubu (central) region of Japan in early September, 2005. On day 1, the subjects walked about 2.5 km. This level of exertion was selected because it closely resembles the average amount of physical activity in a normal working day. This walk was conducted in the forest park during the afternoon. Participants were allowed to rest anywhere and anytime they chose. On day 2, they walked about 2.5 km over two hours both in the morning and afternoon, respectively, in two different forest parks; and on day 3, the subjects finished the trip and returned to Tokyo after blood was drawn and a questionnaire survey was completed. The forests included Japanese cedar (Cryptomeria), Japanese beech, and Japanese oak. Blood was sampled on the second and third days. White blood cell (WBC) counts, NK activity, numbers of NK and T cells, and numbers of granulysin (GRN), perforin, and granzymes A and B (GrA/B)-expressing lymphocytes were measured in the blood samples. The same measurements were made before the trips on a normal working day as a control. Blood was sampled at 8:00 am on all occasions. To control for the effect of alcohol on NK activity, the subjects did not consume alcohol for 2 days before blood was drawn. Phytoncide concentrations in forest air samples were also measured. Walk in forests significantly increased human NK activity and the numbers of NK cells. NK cell activity went up from 17.3% to 26.5% with a 53.2% increase. NK cell numbers went up from 440 to 661 with a 50% increase. It has been reported that NK cells kill tumor or virus-infected cells by the release of perforin, granzymes, and GRN via the granule exocytosis pathway [15]. In order to explore the mechanism of enhancement of NK activity induced by the forest bathing, the effect of forest bathing on the intracellular levels of perforin, GRN, and GrA/B in peripheral blood lymphocytes (PBL) were investigated, and it was found that the forest bathing also significantly increased the numbers of intracellular perforin-, GRN-, and GrA/B-expressing lymphocytes. The presence of anti-cancer protein GRN was up by 48%, GrA by 39%, GrB by 33%, and perforin by 28%. Taken together, these findings indicate that Shinrin-yoku can increase NK activity, and that this effect might be at least partially mediated by increasing the number of NK cells and by the induction of intracellular perforin, GRN, and GrA/B [1]. Han et al., [16] and Tsao et al., [17] also reported that forest bathing increased human NK activity and supported our findings.

#### 1-2 Does a trip to places without forest (a city tourist visit) also increase human NK activity?

Although a forest bathing trip boosted human NK activity, does a trip to places without forest (a city tourist visit) also increase NK activity? Thus, to investigate whether taking a trip (city tourist visit) can also affect human NK activity, eleven healthy male subjects, aged 35–56 years, participated in a three-day/two-night trip to Nagoya city, which is the fourth most populous city in Japan in mid-May, 2006 [8]. Information on the subjects was gathered from a self-administered questionnaire, including age and lifestyle habits as described previously [1]. On the first day, the subjects walked for two hours in the afternoon along a tourist route through a historic district in Nagoya, and then stayed at a hotel also in Nagoya. On the second day, the subjects walked for 2 hours around the Nagoya Baseball Dome in the morning and 2 hours around/in Nagoya Airport in nearby Nagoya city in the afternoon. There are some areas with trees in Nagoya city, but there are almost no trees in the areas visited. The class of hotel was the same and the lifestyle of the subjects during the stays in the hotels was the same for the city and the forest trips. The walking courses in the trip were 2.5 km, which was the same as the previous study [1]. Blood was sampled at 8:00 am on the second and third days after the trip, and three days prior to the trip as a control. WBC counts, NK activity, proportions of NK and T cells, and GRN-, perforin-, and GrA/B-expressing cells in PBL were measured. Adrenaline concentration in urine was also measured. The results showed that the city tourist visit did not increase human NK activity, numbers of NK cells, or the expression of the selected intracellular perforin, GRN, and GrA/B, indicating that increased NK activity during forest bathing trip was not due to the trip itself, but due to forest environments [8].

#### 1-3 How long does the increased NK activity last after a forest bathing trip?

Forest bathing, but not a city trip indeed boosted human NK activity; however, how long does the increased NK activity last after a forest bathing trip? Thus, an investigation was conducted to address this question [8]. Twelve healthy male subjects, aged 35–56 years, were selected from four large companies in Tokyo, Japan. Information on the subjects was gathered from a self-administered questionnaire, including age and lifestyle habits as described previously [1]. The subjects experienced a three-day/two-night trip to three different *Chamaecyparis obtuse* (Japanese cypress, Hinoki in Japanese) forest parks, the birthplace of forest bathing (shinrin-yoku) in Japan, around Agematsu town in Nagano prefecture located in the Chubu (central) region of Japan in early September, 2006. The schedule of the forest bathing trip was similar to that described previously [1]. Blood was sampled at 8:00 am on the second and third days, on days 7 and 30 after the forest bathing trip, and three days prior to the trip as a control. WBC counts, NK activity, proportions of NK and T cells, and GRN-, perforin-, and GrA/B-expressing cells in PBL were measured. Spot urine was sampled at 7:00 am on the second and third days, on days 7 and 30 after the forest bathing trip, and three days prior to the trip as a control. Adrenaline concentration in urine was also measured. The forest bathing trip significantly increased human NK activity, the numbers of NK cells, and the percentages of GRN-, perforin-, and GrA/B-expressing cells in PBL, which confirmed the previous findings [1]. The increased NK activity, number of NK cells, and percentages of GRN-, perforin-, and GrA/B-expressing cells lasted more than 7 days and even for 30 days in the cases of NK activity, the number of NK cells, and GRN- and GrB-expressing cells. These findings indicate that a forest bathing trip increased NK activity, the number of NK cells, and the levels of intracellular perforin, GRN, and GrA/B, and that these effects lasted for at least seven days after the trip, even 30 days [8]. The important finding is that visiting a forest, rather than a city, increases NK activity and the intracellular levels of perforin, GRN, and GrA/B. It is very important in the preventive medicine.

#### 1-4 Effect of Shinrin-yoku on human NK activity in female subjects

Although it has been demonstrated that forest bathing trips enhance human NK activity in male subjects, it still remained to be resolved whether or not a Shinrin-yoku trip also increases NK activity in female subjects. It has been reported that menstrual cycle significantly affects NK activity [18]; therefore, the influence of menstrual cycle on NK activity should be controlled for in experiments with female subjects.

In this study [9], thirteen healthy nurses, aged 25–43 years, professional career 4–18 years, were selected with informed consent. None of the subjects had any signs or symptoms of infectious disease, used drugs that might affect immunological analysis, or were taking any medication at the time of the study. The subjects experienced a three-day/two-night trip to forest fields around Shinano town in Nagano prefecture located in the Chubu (central) region of Japan in early September of 2007. The schedule of the forest bathing trip and blood/urine sampling was similar to that described previously. WBC counts, NK activity, numbers of NK and T cells, and GRN, perforin, and GrA/B-expressing lymphocytes in the blood samples, the concentrations of estradiol and progesterone in serum, the concentrations of adrenaline and noradrenaline in urine were measured. The same control measurements were made before the trip on a normal working day. Blood was sampled at 8:00 am on all days. The concentrations of phytoncides in the forests were also measured. The forest bathing trip significantly increased NK activity and the positive rates of NK, perforin-, GRN-, and GrA/B-expressing cells. The increased NK activity and the positive rates of NK, perforin, GRN, and GrA/B-expressing cells lasted for more than seven days after the trip [9], which confirmed the previous findings in male subjects [8]. Phytoncides, such as α-pinene and β-pinene were detected in forest air. These findings indicate that a forest bathing trip also increased NK activity, the number of NK cells, and the levels of intracellular anti-cancer proteins in female subjects, and that this effect lasted for at least seven days after the trip.

It has been reported that the menstrual cycle and the levels of estradiol and progesterone in serum may affect human NK activity in female subjects [18–20]. To control for the influence of menstrual cycle on NK activity, a questionnaire was administered to obtain information on the menstrual cycle of the subjects. The ratios of subjects who were in the follicular phase during the experiment were 5/13, 6/13, 6/13, 7/13, and 6/13 on the day before the trip, days 1 and 2 during the trip, and days 7 and 30 after the trip, respectively, indicating that there was no significant difference in the proportion of the menstrual cycles of the subjects between the different days. This suggests that the menstrual cycle had a similar influence on the average of NK activity on the different days. In addition, there was no significant difference in the concentrations of estradiol and progesterone in the serum in the days before, during, and after the forest bathing trip, indicating that estradiol and progesterone had a similar effect on NK activity on different days in the subjects in this case [9].

#### 1-5 A day trip to a forest park also increased human NK activity

Although a three-day/two-night Shinrin-yoku enhanced human NK activity, the number of NK cells, and intracellular anti-cancer proteins in lymphocytes, it is not clear whether a day trip to a forest park also increases human NK activity. My research team found that a day trip to a forest park also significantly increased human NK activity, the numbers of NK, perforin, GRN, and GrA/B-expressing cells while significantly decreasing the concentrations of cortisol in the blood and adrenaline in urine in male subjects. The increased NK activity lasted for seven days after the trip. Phytoncides, such as isoprene, α-pinene and β-pinene, were detected in the forest air [21]. In fact, NK activity was increased after day 1 in three-day/two-night forest bathing [1, 8, 9]; therefore, the day trip of forest bathing [21] reproduced the previous findings [1, 8, 9].

The increased NK activity and anti-cancer proteins lasted for more than 7 days, even 30 days after the trip [1, 8, 9, 21, 22]. This suggests that if people take a forest bathing trip once a month, they may be able to maintain a higher level of NK activity. This is very important in terms of health promotion and preventive medicine. NK cells are immune cells and play an important role in defense against bacteria, viruses and tumors. People with higher NK activity showed a lower incidence of cancers, whereas people with lower NK activity showed a higher incidence of cancers [23], indicating the importance of NK cell function on cancer prevention. Therefore, it suggests that Shinrin-yoku may have the preventive effect on cancers.

Many factors, including circadian variation [24], physical exercise [25], and alcohol consumption [25, 26] can affect human NK activity. In order to control for the effect of circadian rhythm on NK activity, blood was sampled at 8:00 am on all days [1, 8, 9, 21, 27]. To control for the effect of physical exercise on NK activity, the walking steps during the trips were limited to the average normal workday distances as monitored by a pedometer. The levels of physical activity among all trips were also matched. To control for the effect of alcohol on NK activity, the subjects did not consume alcohol for two days before blood was drawn during the study period for both trips including before the trips and after the trips on days 7 and 30.

These findings indicate that forest therapy increased NK activity by the following pathways [22].

(1) Shinrin-yoku directly acts on NK cells by phytoncides released from trees and induces increases in the number of NK cells and the levels of intracellular anti-cancer proteins such as perforin, GRN, and GrA/B.
(2) Shinrin-yoku indirectly increases human NK activity, the number of NK cells and the levels of intracellular anti-cancer proteins by reducing stress hormones.

Taken together, because NK cells can kill tumor cells by releasing anti-cancer proteins, such as perforin, GRN, and GrA/B, and forest therapy increases NK activity and the intracellular level of anti-cancer proteins, the above findings suggest that Shinrin-yoku may have a preventive effect on cancer generation and development. My research team also reported that people living in areas with lower forest coverage have significantly higher standardized mortality ratios (SMRs) of cancer than people living in areas with higher forest coverage. There are significant inverse correlations between the percentage of forest coverage and the SMRs of lung, breast, and uterine cancers in females, and the SMRs of prostate, kidney, and colon cancers in males in all prefectures in Japan, even after the effects of smoking and socioeconomic status are factored in, indicating that increased forest coverage may partially contribute to a decrease in mortality due to cancer in Japan [11]. This an ecological study and the evidence level of ecological study is limited.

### 2. Effects of Shinrin-yoku on the nervous system

Forests also regulates the nervous system. The nervous system is made up of the sympathetic nervous system (the ‘fight or flight’ part, which gets your heart going), and the parasympathetic nervous system (the ‘rest and recover’ part, which calms everything down). Common sense tells us that spending time in nature helps us relax and feel calm [28]. Many studies have reported that Shinrin-yoku can increase the activity of parasympathetic nerve and reduce the activity of sympathetic nerve showing relaxing effects (psychologically calming effects) [29–36].

### 3. Effects of Shinrin-yoku on stress hormones

There are three kinds of stress hormones: adrenaline (which mainly indicates mental stress), noradrenaline (which mainly indicates physical stress) and cortisol (which can indicate both) [28]. Adrenaline is released from the adrenal medulla, and the adrenaline level increases under circumstances of novelty, anticipation, unpredictability, and general emotional arousal, whereas noradrenaline is the predominant neurotransmitter released by the sympathetic system, and some of this enters the blood; the level of noradrenaline increases during increased physical activity [37]. Cortisol is released by the hypothalamic-pituitary-adrenal axis in response to stress [31]. My research team has found that Shinrin-yoku and phytoncides can reduce stress hormones, such as adrenaline, noradrenaline and cortisol and may contribute to stress management [8, 9, 21, 27, 29, 36]. In addition, because the effect of forest bathing on adrenaline was greater than that on noradrenaline, the effect on mental stress was greater than on physical stress [3, 9]. Other researchers also reported that Shinrin-yoku reduced cortisol in saliva [31, 32] which supported our findings.

### 4. Effects of Shinrin-yoku on blood pressures and heart rate

Many reports have found that forest environments reduced the levels of blood pressure in middle-aged subjects with high-normal blood pressure [29, 38–42].

Li et al. [29] investigated the effects of forest environments on blood pressure in sixteen male subjects with higher blood pressure without taking antihypertensive drug (mean age: 57.4 ± 11.6 years) after obtaining informed consent. The subjects took day trips to a forest park in the suburbs of Tokyo and to an urban area of Tokyo as a control in September 2010. On both trips, they walked for two hours in the morning and afternoon on a Sunday. Blood and urine were sampled on the morning before each trip and after each trip. Blood pressure was measured on the morning (8:00 am) before each trip, at noon (1:00 pm), in the afternoon (4:00 pm) during each trip, and on the morning (8:00 am) after each trip. Both systolic and diastolic blood pressure levels at noon (1:00 pm) in the forest park were significantly lower than those in the urban area. Moreover, the diastolic blood pressure level in the afternoon (4:00 pm) in the forest park was significantly lower than that in the urban area. However, there was no significant difference in both systolic and diastolic blood pressure levels before walking (8:00 am) between the urban and forest. The reductions in blood pressure after walking in a forest environment were 7 mmHg for both SBP (from 141 to 134 mmHg), and DBP (from 86 to 79 mmHg). This suggests that walking in the forest park, but not in the urban area reduced blood pressure and that forest therapy has a potential preventive effect on hypertension.

Mao et al [39] also found the beneficial effect of forest bathing on blood pressure. In this study, twenty-four elderly patients with essential hypertension were randomly divided into two groups of 12. One group was sent to a broad-leaved evergreen forest to experience a 7-day/7-night trip, and the other was sent to a city area in Hangzhou for control. Blood pressure indicators, cardiovascular disease-related pathological factors including endothelin-1, homocysteine, renin, angiotensinogen, angiotensin II, angiotensin II type 1 receptor, angiotensin II type 2 receptor as well as inflammatory cytokines interleukin-6 and tumor necrosis factor α were detected. As results, subjects who walked in the forest environment showed a significant reduction in blood pressure in comparison to that of the city group. The values for the bio-indicators in subjects exposed to the forest environment were also lower than those in the urban control group and the baseline levels of themselves. They concluded that forest bathing has therapeutic effects on human hypertension by inhibiting the renin-angiotensin system and inflammation. In addition, Ochiai et al. [38] and Yu et al. [40] also found that forest bathing can reduce blood pressure on middle-aged males with high-normal blood pressure. Moreover, Ideno et al [41] conducted a systematic review and meta-analysis including twenty trials involving 732 participants on the effect of Shinrin-yoku on the blood pressure. Both systolic and diastolic blood pressures of the forest environment was significantly lower than that of the non-forest environment showing a significant effect of Shinrin-yoku on reduction in blood pressure. Yau and Loke [42] also reviewed the physiologically and psychologically relaxing effects of forest bathing on middle-aged and elderly people with pre-hypertension and hypertension, indicating that forest bathing shows preventive effect on hypertension. Li et al [36] also found that forest bathing reduced heart rate in middle-aged males.

Taken together, Shinrin-yoku reduces blood pressure by the following mechanisms:

1) Shinrin-yoku reduces blood pressure by reducing stress hormone levels, such as urinary adrenaline, urinary noradrenaline [8, 9, 21, 29, 36], salivary cortisol [31, 32], and blood cortisol [21] levels. It is well known that stress hormones such as adrenaline, noradrenaline and cortisol increase blood pressure level.
2) Shinrin-yoku reduces blood pressure by reducing sympathetic nerve activity and by increasing parasympathetic nerve activity. Sympathetic nerve activity can be determined by measuring the levels of urinary adrenaline and/or noradrenaline [8, 9, 43], and there are significant correlations between blood pressure and urinary adrenaline and noradrenaline levels [44]. In addition, many studies [31–33, 36] reported that forest viewing and walking in forests significantly reduced sympathetic nerve activity and increased parasympathetic nerve activity compared to performing the same activities in an urban environment.
3) Shinrin-yoku reduces blood pressure by inhibiting the renin-angiotensin system [39].

### 5. Potential preventive effects of Shinrin-yoku on depressed states

Shinrin-yoku can reduce the symptoms for anxiety, depression, anger, fatigue and confusion and increased the vigor in the Profile of Mood States (POMS) test in both male and female subjects [1, 3, 7, 9, 21, 29–36, 45]. In addition, forest bathing is particularly effective against mental stress (mental fatigue) [3]. Li et al [46] reported that Shinrin-yoku significantly increased level of serotonin in serum, and significantly increased the score for vigor and decreased the score for fatigue in the POMS test. Furuyashiki et al [47] conducted a comparative study of the physiological and psychological effects of a day-long session of Shinrin-yoku on working age people with and without depressive tendencies and demonstrated significant positive effects on mental health, especially in those with depressive tendencies. These studies suggest Shinrin-yoku has a preventive effect on subjects in a depressed state.

### 6. Effect of Shinrin-yoku on sleep

Three studies investigated the effect of Shinrin-yoku on sleep [3, 46, 48, 49]. We previously found that Shinrin-yoku significantly increased sleep time in middle-aged male office workers [3]. Recently, my research team found that Shinrin-yoku significantly improved the sleepiness on rising and the feeling refreshed (recovery from fatigue) assessed by the Oguri-Shirakawa-Azumi sleep inventory MA version (OSA-MA), indicating that Shinrin-yoku may improve sleep quality [46, 48]. Morita et al [49] also reported that two hours of forest walking improved nocturnal sleep conditions for individuals with sleep complaints, possibly as a result of exercise and emotional improvement.

### 7. Effect of Shinrin-yoku on adiponectin

Adiponectin is a serum protein hormone specifically produced by adipose tissue. Studies have shown that lower blood adiponectin concentrations are associated with several metabolic disorders, including obesity, type 2 DM (diabetes mellitus), cardiovascular disease, and metabolic syndrome. Recent studies have suggested that adiponectin shows anti-tumorigenesis activity in several cancers, including prostate, breast, endometrial, brain, and colon cancer [50, 51]. My research team found that Shinrin-yoku can increase the level of serum adiponectin [29, 36]. However, there are only two studies on the effect of forest bathing on adiponectin so far; therefore, further studies on this topic are needed.

### 8. Effect of Shinrin-yoku on dehydroepiandrosterone sulfate (DHEA-S)

Levels of DHEA and DHEA-S, the major secretory products of the adrenal gland, decline dramatically with age, concurrent with the onset of degenerative changes and chronic diseases associated with aging [52, 53]. Epidemiological evidence in humans suggests that DHEA-S has cardioprotective, antiobesity, and antidiabetic properties [53]. My research team found that Shinrin-yoku significantly increase serum dehydroepiandrosterone sulfate (DHEA-S) levels [29]. On the other hand, Kim et al [54] reported that a Forest Healing Program in a Korean forest reduced DHEA-S levels. Therefore, the effect of forest bathing on DHEA-S needs further research.

### 9. Shinrin-yoku may apply to rehabilitation medicine

Depression is reportedly the most common mental disorder following stroke, with an incidence ranging from 10 to 64%. Poststroke depression has an adverse effect on functional recovery and increases the mortality rate [55, 56]. In addition, nearly one-third of patients suffer from depression and more than one-quarter of patients suffer from PTSD after an acute orthopaedic injury [57]. Based on the above background, the prevention of depression in rehabilitation hospital is a big challenge in the world. It is urgent to establish preventive measures against depression; however, effective prevention methods have not been established at present. My research team previously found that the Shinrin-yoku significantly increased the score for vigor and decreased the scores for anxiety, depression, anger, fatigue, and confusion in the POMS test accompanied by reductions in urinary adrenaline and noradrenaline concentrations in both males and females, suggesting that forest bathing may have potential preventive effects on depression. Moreover, we also found that walking in city parks in Tokyo reduces the negative emotions such as tension–anxiety, anger, depression, fatigue and confusion and increase in feelings of vigor in the POMS test and showed the relaxing effect both in male and female subjects [1, 3, 9, 21, 29–36, 45, 48]. These findings suggest that Shinrin-yoku may have a potential preventive effect on depressive status. Thus, my research team investigated the relaxing effects of Shinrin-yoku on rehabilitation patients by walking in a Japanese garden for applying the forest bathing in stress management and prevention of depression in patients in rehabilitation hospitals to improve the rehabilitative effect. My research team found that Shinrin-yoku in a Japanese garden reduces the scores of anxiety, depression, anger, fatigue and confusion, whereas increase the score of vigor in patients in a rehabilitation hospital suggesting that Shinrin-yoku may show apply to the stress management and depression prevention [48, 58]. Some patients who have experienced stroke and acute orthopedic injuries and cannot walk also can enjoy Shinrin-yoku in a wheelchair.

### 10. Effect of phytoncides released from trees on human health

Why did the forest environment affect human health? What kind of factors in the forest environment contribute to beneficial effects on human health? The quiet atmosphere, beautiful scenery, mild climate, special good smell, and fresh, clean air in forests contribute to the effects. It is the total effect from all five senses: senses of sight, smell, hearing, touch and taste. In fact, sense of smell by breathing in volatile organic substances, called phytoncides from trees, such as α-pinene and limonene has a bigger effect [27, 59]. My research team found that phytoncides released from trees significantly increased human NK activity and the intracellular levels of perforin, GrA, and GRN in human NK cells both in vitro [59] and in vivo [27]. Phytoncide exposure significantly decreased the concentrations of adrenaline and noradrenaline in urine, indicate that phytoncide exposure and decreased stress hormone levels may partially contribute to increased NK activity [27].

### 11. The potential preventive effect of Shinrin-yoku on non-communicable diseases

It has been reported that stress may induce and/or exacerbate many non-communicable diseases, such as cancers, hypertension, ischemic heart disease, gastrointestinal ulcer, and depression [7]. Shinrin-yoku can reduce stress hormone levels, such as urinary adrenaline, urinary noradrenaline [8, 9, 21, 29, 36], salivary cortisol [31, 32], and blood cortisol [21] levels suggesting that Shinrin-yoku may have preventive effects on non-communicable diseases mediated by reducing the stress hormones. It has been reported that Shinrin-yoku reduces blood pressure and heart rate showing potential preventive effect on hypertension [29, 36, 38, 39]. It also has been reported that Shinrin-yoku effectively decreases blood glucose levels in type 2 DM (diabetes mellitus) patients and shows preventive effect on type 2 DM [60]. In addition, Shinrin-yoku shows potential preventive effects on depression by reducing stress hormones [8, 9, 21, 29, 31, 32, 36], by reducing negative emotions such as anxiety, depression, anger, fatigue, confusion, and by increasing the level of serotonin in serum and the positive feelings such as vigor [1, 3, 7, 21, 29–36, 45, 46]. Shinrin-yoku-induced increases of the level of serum adiponectin [29, 36] and DHEA-S [29] also contribute to this effect. Moreover, Shinrin-yoku may have preventive effects on cancers by increasing anticancer proteins in NK cells, such as perforin, granulysin and granzymes [1, 8, 9, 21, 27].

### 12. Potential preventive effects of Shinrin-yoku on COVID-19

Elderly people, patients with underlying diseases such as diabetes, hypertension, heart diseases and respiratory diseases are easy to develop COVID-19 and become more severe, and the mortality rate is also higher because of the reduced immune function in these patients [61]. Therefore, immune function is very important to prevent COVID-19. Shinrin-yoku may have preventive effect on COVID-19 by boosting immune function [22]. Mental stress and various mental disorders due to “lockdown” and “isolation” are also major social problems [62]. Shinrin-yoku reduces the negative emotions, mental stress and stress hormones, and increases vigor [1, 3, 8, 9, 29, 36]. In fact, my research team has found that virtual exposure to forest environments based on audio-visual stimuli brought by a short computer video showing forest environments, with an urban video as a control showed effective to reduce negative emotions such as anxiety in people forced by lockdown in limited spaces in Italy during COVID-19 pandemic [62]. Kim et al also reported the positive effects of a Forest Healing Program in a Korean forest on motional stress and sleep quality for exhausted medical workers during the COVID-19 Outbreak in Korea [54]. Therefore, Shinrin-yoku may have preventive effect on COVID-19-induced mental stress and mental disorders. Shinrin-yoku also has preventive effects on hypertension and heart diseases [29, 36] to prevent COVID-19.

Taken together, Shinrin-yoku will play a very important role on the preventive of COVID-19 by boosting immune function and by reducing mental stress in post-COVID-19 health management and disease prevention.

Wen et al [63] conducted a systematic review in studies on forest bathing (Shinrin-yoku) and concluded that forest bathing (Shinrin-yoku) might have the following merits: remarkably improving cardiovascular function, hemodynamic indexes, neuroendocrine indexes, metabolic indexes, immunity and inflammatory indexes, antioxidant indexes, and electrophysiological indexes; significantly enhancing people’s emotional state, attitude, and feelings towards things, physical and psychological recovery, and adaptive behaviors; and obvious alleviation of anxiety and depression.

## Forest Medicine in the future

Based on the above background, I would like to propose the following international collaborations on Forest Medicine in the future.

1. To expand the philosophy and concept of Forest Medicine over the world.
2. To verify the preventive effects of Forest Medicine on non-communicable diseases in the world.
3. To establish an international accreditation system for Forest Medicine specialist and Forest Therapist.
4. To establish the Shinrin-yoku/Forest bathing as a treatment for some non-communicable diseases.
5. To incorporate the Forest Medicine into Rehabilitation Medicine.

## Declaration

### Availability of data and material

The datasets used and analyzed during the current study are available on reasonable request to the corresponding author.

### Fundings

These studies were conducted and supported by the following fundings.

1. A research project for utilizing advanced technologies in agriculture, forestry and fisheries of Japan (2004–2006).
2. Grants-in-Aid for Scientific Research from the Ministry of Education, Culture, Sports, Science and Technology (No. 16107007).
3. Research projects from the National Land Afforestation Promotion Organization
4. A research project from the Forestry and Forest Products Research Institute (2009–2010)
5. A research project from Japan Society for the Promotion of Science (JSPS) (2011–2012).
6. A research project from the Ministry of Agriculture, Forestry and Fisheries Strategic Innovation Creation Program
7. Research projects from the Vehicle Racing Commemorative Foundation of Japan (2013–15, 2019).

### Authors’ information

Qing Li, MD, PhD, clinical professor, Department of Rehabilitation Medicine, Graduate School of Medicine, Nippon Medical School, Tokyo, Japan, Vice-President & Secretary General of International Society of Nature and Forest Medicine (INFOM), President of Japanese Society of Forest Medicine, Director of Forest Therapy Society (NPO corporation).
